# Patient Priorities-Aligned Intervention Decisions (PPAID) Approach

**DOI:** 10.1093/geroni/igaf122.2024

**Published:** 2025-12-31

**Authors:** Aanand Naik

**Affiliations:** UTHealth Houston, Houston, Texas, United States

## Abstract

Decision-making for surgery and procedures is challenging for older adults with multiple chronic conditions (MCCs) due to uncertain benefits and heightened risks. These individuals vary in the health outcomes they desire most, necessitating discussions that go beyond ‘fixing’ a clinical problem. However, intervention decisions usually do not consider the likelihood of achieving patient-identified outcome goals. The Patient Priorities Care (PPC) approach reduces treatment burden and improves alignment with patient goals and priorities in managing chronic conditions. We adapted PPC principles for discrete intervention decisions into a tool that can be completed with clinician guidance or self-directed with probes. The Patient Priorities-Aligned Intervention Decisions (PPAID) approach was developed through an iterative process incorporating clinician expert and patient input. Unlike traditional decision aids, PPAID centers discussions on specific, realistic and actionable goals rather than procedural choices, ensuring decisions align with patient outcome goals. The PPAID approach builds from four recurring goal categories that broadly capture the outcomes that patients desire from surgery. These categorical goals anchor subsequent deliberations, resulting in the identification of a specific and realistic outcome goal for the given discrete intervention. Future research will evaluate tool feasibility, acceptability, and effectiveness in clinical settings using an artificial intelligence enabled interface. Metrics will include pre-procedure goal agreement, decision regret, and patient satisfaction. Integrating the tool into clinical workflows is crucial for its adoption. By emphasizing goal identification and deliberation, PPAID approach supports age-friendly, patient goals-aligned care as required by the new CMS age-friendly measure.

